# *H. pylori* infection and extra-gastroduodenal diseases

**DOI:** 10.1186/s12929-018-0469-6

**Published:** 2018-08-29

**Authors:** Feng-Woei Tsay, Ping-I Hsu

**Affiliations:** 1 Division of Gastroenterology and Hepatology, Department of Internal Medicine, Kaohsiung Veterans General Hospital and National Yang-Ming University, 386 Ta Chung 1st Road, Kaohsiung, 813 Taiwan, Republic of China; 2 Cheng Shiu University, Kaohsiung, Taiwan, Republic of China

**Keywords:** *Helicobacter pylori*, Iron deficiency anemia, Idiopathic thrombocytopenic purpura and vitamin B12 deficiency

## Abstract

*Helicobacter pylori* infection is the principal cause of peptic ulcer disease, gastric adenocarcinoma and gastric mucosa-associated lymphoid tissue lymphoma. Recent studies have shown that it may interfere with many biological processes and determine or influence the occurrence of many diseases outside the stomach. Currently, the role of *H. pylori* in idiopathic thrombocytopenic purpura and iron deficiency anemia is well documented. Emerging evidence suggests that it may also contribute to vitamin B12 deficiency, insulin resistance, metabolic syndrome, diabetes mellitus and non-alcoholic liver disease. Additionally, it may increase the risk of acute coronary syndrome, cerebrovascular disease, neurodegenerative disease and other miscellaneous disorders. Different pathogenic mechanisms have been hypothesized, including the occurrence of molecular mimicry and the induction of a low-grade inflammation. This review summarizes the results of the most relevant studies on the extra-gastroduodenal manifestations of *H. pylori* infection.

## Background

*Helicobacter pylori* infection is the principal cause of chronic gastritis, gastric ulcer, duodenal ulcer, gastric adenocarcinoma and gastric mucosa-associated lymphoid tissue lymphoma [1, 2]. In recent decades, many articles have published on the fascinating topic of extragastroduodenal manifestations of *H. pylori* infection, including hematological, metabolic, cardiovascular, neurodegenerative and allergic disorders [3–13]. Different pathogenic mechanisms have been hypothesized, including the occurrence of molecular mimicry and the induction of a low-grade inflammation. Indeed, *H. pylori* infection is a very good model for studying host-bacterial interactions and very attractive for those interested in the role of gut microbiota in health and diseases. Here, we summarize the results of the most relevant studies on the extragastroduodenal manifestations of *H. pylori* infection.

## Iron deficiency anemia

The link between Iron deficiency anemia (IDA) and *H. pylori* infection was reported firstly in 1991 by Blecker et al., who cured IDA of a 15 year-old female presenting with anemia-related syncope and *H. pylori*-induced chronic active hemorrhagic gastritis by eradication therapy without iron supplements [14]. The association of *H. pylori* infection with unexplained IDA has been proven in adult and pediatric populations [15, 16] though a few investigations didn’t show this link [17, 18]. Recently, Qu et al. conducted a meta-analysis of 15 case-control studies to investigate the relation between *H. pylori* infection and IDA [19]. *H. pylori* infection was diagnosed by endoscopy and histological examination in five studies, in which patients with peptic ulcer disease and gastric cancer were not included. The other 10 studies confirmed *H. pylori* infection by serology or urea breath test. The data showed an increased risk of IDA in patients with *H. pylori* infection with an odds ratio (OR) of 2.2 (95% confidence interval [CI]:1.5–3.2) [19]. Several works also demonstrated recovery from IDA by successful eradication of *H. pylori* without iron supplements [20]. Yuan et al. performed a meta-analysis of 16 randomized controlled trials involving 956 patients to assess the impact of *H. pylori* eradication therapy on IDA [21]. In this work, the diagnosis of *H. pylori* infection was based on rapid urease test or histology in eight studies, in which patients with peptic ulcer disease were excluded. The other eight studies confirmed *H. pylori* infection by urea breath test. The follow-up time in these studies ranged from 1 to 3 months. The difference from baseline to endpoint of hemoglobin, serum iron, and serum ferritin in the meta-analysis was statistically significantly different between anti-*H. pylori* treatment plus oral iron and oral iron alone (differences: Hb, 1.48 g/dL; serum iron: 1.15 mol/L; serum ferritin, 1.84 ng/mL) [21].

*H. pylori* causes IDA by several mechanisms. First, increased iron loss can be due to hemorrhagic gastritis, peptic ulcer disease and gastric adenocarcinoma [22]. Second, CagA protein of *H. pylori* has been shown to participate in iron acquisition from interstitial holotransferrin [23]. Iron uptake by *H. pylori* is enhanced during the growth of the bacteria [24]. Third, *H. pylori*-related corporal gastritis may decrease acid secretion due to gland atrophy and results in the reduction of iron absorption from diet [25].

In summary, the association of *H. pylori* and IDA has been conclusively proven in numerous studies. Current international and national guidelines recommend eradication of *H. pylori* infection in patients with unexplained IDA [26, 27].

## Immune thrombocytopenic purpura

Gasbarrini et al. reported the first case of *H. pylori* infection associated with immune thrombocytopenic purpura (ITP) in 1998 [28]. An observation study from Japan also found a good platelet response in ITP patients treated by *H. pylori* eradication [29]. A randomized controlled trial by Brito et al. revealed that *H. pylori* eradication resulted in a significant platelet response in children and adolescents affected by ITP [30]. The role of *H. pylori* infection in ITP has also been confirmed by several other studies [31, 32]. Nonetheless, some studies from countries with low prevalence of infection, like France and the United States, did not find the link between *H. pylori* infection and ITP [33, 34]. Recently, Stasi et al. conducted a meta-analysis of 25 studies to investigate the impact of anti-*H. pylori* therapy on ITP [34]. The assessing time for platelet response ranged from one to six months. The data showed that the rates of complete response (platelet count ≧ 100 × 10^9^/L) and overall response (platelet count ≧ 30 × 10^9^/L and at least doubling of the basal count) after successful eradication of *H. pylori* were 42.7 and 50.3%, respectively [35]. The predictors of a good response to eradication therapy were countries with higher prevalence of *H. pylori* infection (such as Japan and Italy) and patients with milder degree of thrombocytopenia [35]. In the majority of ITP patients responding to anti-*H. pylori* therapy, the durability of platelet response is more than 7 years, indicating the disease is cured [36]. Another meta-analysis by Arnold et al. performed a meta-analysis to determine the effect of *H. pylori* eradication therapy in patients with ITP by comparing the platelet response in ITP patients with and without *H. pylori* infection [37]. The odds of achieving a platelet count response following eradication therapy were 14.5 higher (95% CI: 4.2 to 83.0) in patients with *H. pylori* infection than in those without infection (response rate: 51.2% vs. 8.8%). These findings strengthen the causal association between *H. pylori* infection and ITP. Several mechanisms regarding *H. pylori*-associated ITP have been proposed [38]. One intriguing hypothesis concerning molecular mimicry is that cross-reactive antibodies are produced that react both *H. pylori* components and platelet surface antigens. Takahashi et al. showed that platelet elutes from *H. pylori*-infected ITP patients recognized CagA protein in immunoblots, but those from *H. pylori*-infected non-ITP patients did not [39]. Bai et al. also reported that monoclonal antibodies generated against *H. pylori* urease B react with GP IIb/IIIa expressed on the platelet surface [40]. While these findings suggest molecular mimicry between *H. pylori* components and platelet surface antigens, the exact pathogenic roles of these cross-reactive antibodies remain obscure. In another potential mechanism, *H. pylori* infection may alter Fcγ receptor balance of moncytes/macrophages and induce autoantibody formation. A recent study showed that the FcγR II B expression on circulating monocytes was down-regulated in *H. pylori*-infected ITP patients [41]. Therefore, *H. pylori* may alter Fcγ receptor balance of moncytes/macrophages through downregulation of the inhibitory receptor FcγR II B.

In conclusion, many studies support the association between *H. pylori* infection and ITP. Current international and national guidelines recommend that *H. pylori* infection should be sought and treated in patients with ITP [27].

## Vitamin B12 deficiency

The link between vitamin B12 deficiency and *H. pylori* infection was reported firstly in 1984 by O’Connor et al. who showed Campylobacter-like organisms in patients with type A gastritis and pernicious anemia [42]. Studies have demonstrated a link between chronic *H. pylori* infection and malabsorption of vitamin B12 [43]. Sarari et al. showed that vitamin B12 deficiency was present in 67.4% (29/43) of the patients with *H. pylori* infection [44]. Shuval-Sudai et al. found a higher prevalence of *H. pylori* infection in patients at the lower end of the normal range of serum vitamin B12 levels [45]. However, most studies regarding the association between vitamin B12 and *H. pylori* infection focus on testing *H. pylori* status and measuring serum levels of vitamin B12. No adequate interventional studies proving the effect of anti-*H. pylori* therapy on vitamin B12 deficiency exist.

## Metabolic syndrome and diabetes mellitus **(DM)**

Many epidemiological studies have supported a link between insulin resistance, metabolic syndrome and *H. pylori* infection [46, 47]. Chen et al. demonstrated that *H. pylori*-infected subjects had a higher prevalence of metabolic syndrome than those without *H. pylori* infection [48]. Additionally, Yang et al. showed a significant association between *H. pylori* infection and DM [49]. Similar results were also observed by other investigators [50]. Furthermore, Horikawa et al. revealed that *H. pylori* infection worsened glycemia control in diabetic patients [51]. Polyzos et al. conducted a systemic review including nine studies and showed a trend toward a positive association between *H. pylori* infection and insulin resistance [47]. In contrast, several studies did not find the link between *H. pylori* infection and insulin resistance or metabolic syndrome [52]. Naja et al. showed no association between *H. pylori* infection and metabolic syndrome in a Lebanese population [53]. A meta-analysis of 18 studies found no strong correlation between *H. pylori* infection and serum concentrations of total cholesterol and triglyceride [54]. Wada et al. also found that successful eradication of *H. pylori* could not improve glucose control of DM in Japanese patients [55]. Furthermore, a recent randomized controlled trial involving 49 *H. pylori*-infected subjects in a prediabetes stage showed that *H. pylori* eradication resulted in an increased Homeostatic model assessment of insulin resistance (HOMA-IR) [56].

Several studies reported a reverse link between *H. pylori* infection and obesity [57–60]. A case-control study from Taiwan demonstrated an inverse relationship between morbid obesity and *H. pylori* seropositivity [57]. An ecological study also showed an inverse correlation between *H. pylori* prevalence and rate of overweight/obesity in countries of the developed world [58]. However, a large case-control study including 8820 participants from China showed body mass index was significantly and positively associated with *H. pylori* infection [59]. An intervention trial demonstrated serum ghrelin concentrations were inversely related to the severity of *H. pylori*-associated gastritis in prepubertal children [60]. Eradication of *H. pylori* infection resulted in a significant increase in body mass index along with a significant decrease in circulating ghrelin levels and an increase in leptin levels [60].

In summary, the issue of the association between *H. pylori* infection and metabolic syndrome or DM remains contradictory.

## Nonalcoholic fatty liver disease (NAFLD)

A cohort study by Kim et al. demonstrated that the subjects with *H. pylori* infection had a higher incidence of NAFLD than those without infection (hazard ratio: 1.21 [95% CI: 1.1–1.3]) [61]. Polyzos et al. also revealed that patients with NAFLD had higher anti-*H. pylori* IgG titers, together with lower circulating adiponectin and higher tumor necrosis factor-α levels, compared to non-NAFLD subjects [62]. However, opposite results from Korea and Japan showed no association between *H. pylori* infection and NAFLD [63, 64]. Recently, a meta-analysis demonstrated a significantly increased risk of NAFLD in patients with *H. pylori* infection [65]. Nonetheless, the mechanism underlying the association between *H. pylori* infection and NAFLD remains unclear, and interventional studies proving the effect of anti-*H. pylori* therapy on NAFLD are fairly limited.

In summary, the association between *H. pylori* infection and NAFLD remains contradictory.

## Coronary artery disease (CAD)

Mendall et al. first showed a link between *H. pylori* and CAD in 1994 [66]. Several studies reported that CagA-postive strains of *H. pylori* were associated with atherosclerosis [67–69]. Al-Ghamdi et al found that *H. pylori* plays an important role in the development of CAD by altering the lipid profile and enhancement of chronic inflammation [70]. Figura et al. also revealed that CagA-postive strains of *H. pylori* were associated with high serum levels of interleukin-6 and B-type natriuretic peptide in patients with CAD [71]. A nationwide retrospective cohort study demonstrated that *H. pylori* infection increased the risk of acute coronary syndrome [72]. In addition, a meta-analysis of 26 studies involving more than 20,000 patients also showed a significant association between *H. pylori* infection and the risk of myocardial infarction (OR: 2.10; 95% CI: 1.8–2.5) [73]. However some studies from Indian and German did not find the association between *H. pylori* and CAD [74, 75]. Additionally, there are still no interventional studies proving the beneficial effect of *H. pylori* eradication in decreasing the incidence of CAD.

There are several proposed mechanisms underlying the association between *H. pylori* infection and CAD. *H. pylori* has been detected in human carotid atherosclerotic plaques [76]. Oshima et al. demonstrated the association of *H. pylori* infection with systemic inflammation and endothelial dysfunction in healthy male subjects [77]. They proposed that *H. pylori* infection may cause atherogenesis through persistent low-grade inflammation. Recently, molecular mimicry between CagA antigen of *H. pylori* and atherosclerotic plaque peptides has also been proposed as a possible mechanism [78].

In conclusion, there is controversial evidence linking *H. pylori* infection and CAD. No adequate interventional trials demonstrating a lower incidence of CAD as a result of anti-*H. pylori* therapy exit.

## Cerebrovascular disease

Wincup et al. first reported a link between *H. pylori* infection and stroke in 1996 (OR = 1.57, 95% CI 0.95 to 2.60) [79]. A Mexican study found that levels of antibodies to *H. pylori* predict incident stroke in fully adjusted models (OR: 1.58; 95% CI: 1.1 to 2.3) [80]. Recently, Wang et al. performed a meta-analysis of 4041 Chinese patients, and found an association between *H. pylori* infection and non-cardioembolic stroke [81]. However, a cohort study of 9895 cases from the United States found a reverse link between *H. pylori* infection and stroke mortality, and this reverse association was stronger for *H. pylori* cagA positivity [82]. In summary, there is controversial evidence linking *H. pylori* infection and cerebrovascular disease.

## Other miscellaneous disorders

Some studies also disclosed the relationship of *H. pylori* with dementia and Alzheimer’s disease (AD) [83, 84]. A study in Greece by Kountouras et al. found higher prevalence of *H. pylori* infection in patients with AD than in the control group [85]. Hung et al. designed a study for the relationship between *H. pylori* infection and non-Alzheimer’s dementia (non-AD) using a nationwide population-based dataset in Taiwan, and found that patients with *H. pylori* infection were 1.6-fold more likely to develop non-AD than those without infection [83]. A retrospective cohort study using nationwide database in Taiwan showed that eradication of *H. pylori* was associated with a decreased progression of dementia as compared to no eradication of *H. pylori* in AD patients with peptic ulcers [86]. However, further prospective randomized control trials are needed to clarify these findings.

The inverse relationship between *H. pylori* infection and allergic asthma has been reported. A meta-analysis by Zhou et al.*..* in 2013 found lower prevalence rate of *H. pylori* infection in patients with allergic asthma [87]. Higher prevalence rate of *H. pylori* infection has been found in cirrhotic patients with hepatoencephalopathy than in those without hepatoencephalopathy [88]. Jaing et al also showed the association of *H. pylori* infection with elevated blood ammonia levels in cirrhotic patients [89]. Several studies have also reported that *H. pylori* infection increases the risk of colon adenocarcinoma and adenoma [90–92]. Recently, an association between *H. pylori* infection and chronic spontaneous urticaria has been reported but remains controversial. Fukuda et al. demonstrated a significant improvement of chronic spontaneous urticaria by anti-*H. pylori* therapy in Japanese patients [93]. This work was consistent with a systemic review of 10 studies by Federman et al. [94]. However, Moreira et al. did not find the association between *H. pylori* infection and chronic spontaneous urticaria [95].

In summary, there are still controversial evidences linking *H. pylori* infection and aforementioned miscellaneous disorders. Adequate interventional trials are needed to clarify these associations.

## Conclusions

**Fig. 1:**
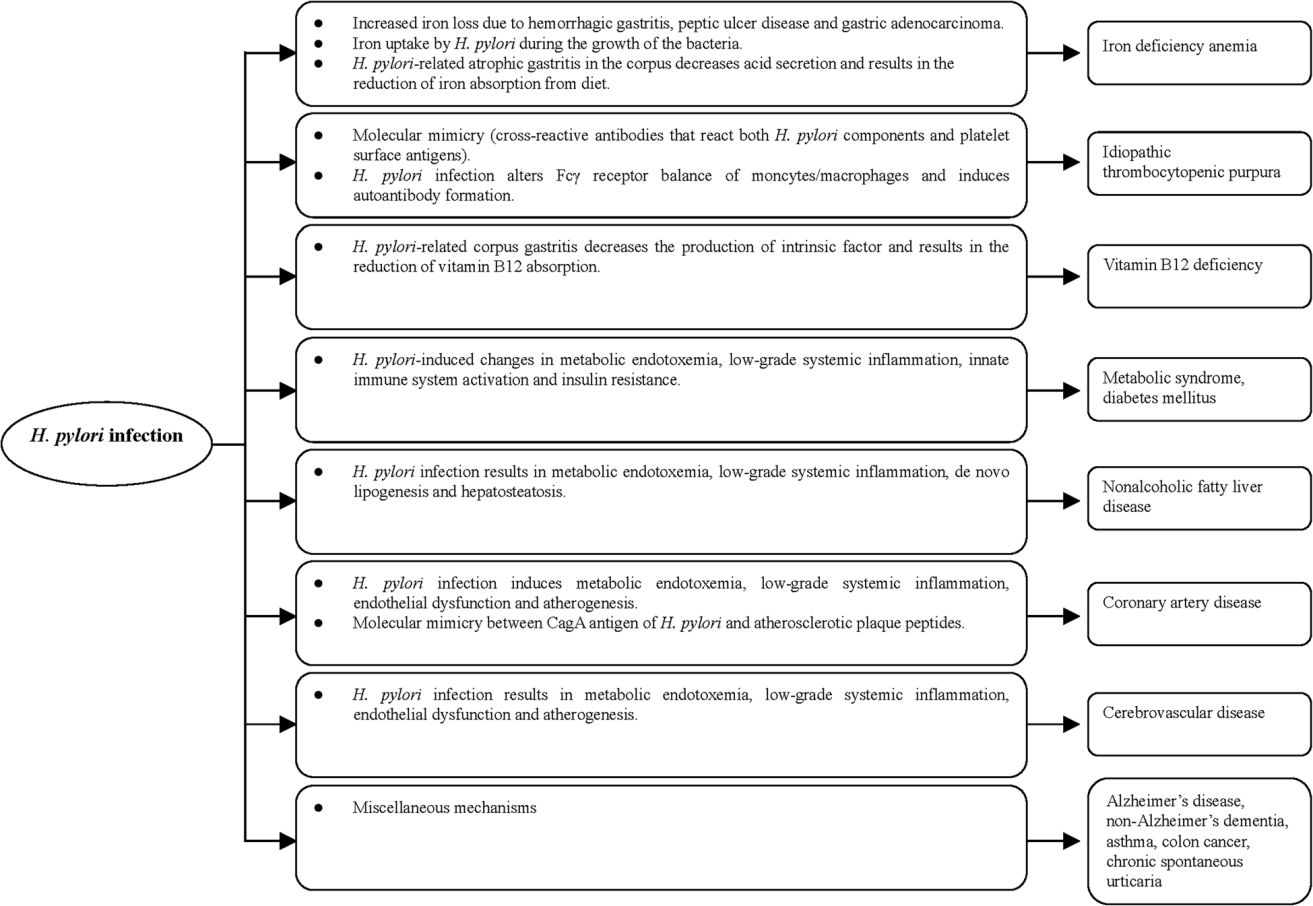
The possible mechanism for the association between *H. pylori* infection and extra-gasroduodenal diseases

Recent studies have shown that *H. pylori* may interfere with many biological processes and determine or influence the occurrence of many diseases outside the stomach (Table 1 and Fig. 1). Currently, its role in ITP and IDA is well documented. Emerging evidence suggests that it may also contribute to vitamin B12 deficiency, insulin resistance, metabolic syndrome, diabetes mellitus and non-alcoholic liver disease. Additionally, it may also increase the risk of acute coronary syndrome, cerebrovascular disease, and neurodegenerative disease, *H. pylori* infection is a perfect model for the study of interplay between human beings and bacteria. Further studies are mandatory to clarify the pathogenesis of extragastroduodenal diseases induced by *H. pylori* infection.

**Table 1:** The relevant studies on the associations between *H. pylori* infection and extra-gastroduodenal diseases

	Extra-gastroduodenal disease	Key evidences	Conclusion
1	Iron deficiency anemia (IDA)	Pros: 1. Qu et al. [19]: an increased risk of IDA in patients with *H. pylori* infection (meta-analysis of case-control studies). 2. Yuan et al. [21]: Eradication of *H. pylori* could improve the levels of hemoglobin and serum ferritin in patients with IDA (meta-analysis of intervention trials). Cons: 1. Sandstrom et al. [18]: no association between *H. pylori* infection and IDA in female adolescents (case-control study).	Eradication of *H. pylori* infection is recommended for patients with unexplained IDA.
2	Immune thrombocytopenic purpura (ITP)	Pros: 1. Stasi et al. [35]: The overall response rate of increased platelet count was 50.3% after successful eradication of *H. pylori* in ITP patients (meta-analysis of intervention trials). 2. Arnold et al. [37]: The odds of achieving a platelet count response following eradication therapy were 14.5 higher in ITP patients with *H. pylori* infection than in those without infection (response rate: 51.2% vs. 8.8%) (meta-analysis of intervention trials). Cons: 1. Michel et al. [34]: Seroprevalence of *H. pylori* in patients with ITP was not significantly different from that in control subjects (case-control study).	*H. pylori* infection should be sought and treated in patients with ITP.
3	Vitamin B12 deficiency	Pros: 1. Sarari et al. [44]: There was significant association between the presence of *H. pylori* infection and vitamin B12 deficiency (case-control study). 2. Shuval-Sudai et al. [45]: Prevalence of *H. pylori* seropositivity was significantly higher among subjects with borderline (> 145–180 pg/mL) or low normal (> 180–250 pg/mL) vitamin B12 levels than among those with vitamin B12 > 250 pg/mL (case-control study).	*H. pylori* infection is associated with vitamin B12 deficiency.
4	Metabolic syndrome and diabetes mellitus (DM)	Pros: 1. Chen et al. [48]: *H. pylori*-infected subjects had a higher prevalence of metabolic syndrome than those without *H. pylori* infection (case-control study). 2. Yang et al. [49]: *H. pylori* infection was associated with risk of DM (case-control study). Cons: 1. Naja et al. [53]: no association between *H. pylori* infection and metabolic syndrome (case-control study). 2. Wada et al. [55]: The eradication of Helicobacter pylori does not affect glycemic control in Japanese subjects with type 2 diabetes (intervention trial).	The association between *H. pylori* infection and metabolic syndrome or DM is contradictory.
5	Nonalcoholic fatty liver disease (NAFLD)	Pros: 1. Kim et al. [61]: The subjects with *H. pylori* infection had a higher incidence of NAFLD than those without infection (cohort study). 2. Wijarnpreecha et al. [65]: a significantly increased risk of NAFLD in patients with *H. pylori* infection (meta-analysis of case-control studies). Cons: 1. Okushin et al. [63]: no association between *H. pylori* infection and NAFLD (case-control study).	The association between *H. pylori* infection and NAFLD remains contradictory.
6	Coronary artery disease (CAD)	Pros: 1. Yu et al. [73]: significant association between *H. pylori* infection and the risk of myocardial infarction (meta-analysis of case-control studies). Cons: 1. Schottker et al. [75]: no association between *H. pylori* infection and the risk of CAD (cohort study).	The association between *H. pylori* infection and CAD is contradictory.
7	Cerebrovascular disease	Pros: 1. Wang et al. [81]: significant association between *H. pylori* infection and non-cardioembolic stroke (meta-analysis of case-control studies). Cons: 1. Chen et al. [82]: a reverse link between *H. pylori* infection and stroke mortality (cohort study).	There is controversial evidence linking *H. pylori* infection and cerebrovascular disease.

## Abbreviations

AD: Alzheimer’s disease
CI: Confidence interval
DM: Diabetes mellitus
IDA: Iron deficiency anemia
ITP: Immune thrombocytopenic purpura
NAFLD: Nonalcoholic fatty liver disease
OR: Odds ratio
